# Rose Bengal Acetate PhotoDynamic Therapy (RBAc-PDT) Induces Exposure and Release of Damage-Associated Molecular Patterns (DAMPs) in Human HeLa Cells

**DOI:** 10.1371/journal.pone.0105778

**Published:** 2014-08-20

**Authors:** Elisa Panzarini, Valentina Inguscio, Gian Maria Fimia, Luciana Dini

**Affiliations:** 1 Department of Biological and Environmental Science and Technology (Di.S.Te.B.A.), University of Salento, Lecce, Italy; 2 National Institute for Infectious Diseases IRCCS “L. Spallanzani,” Rome, Italy; Wellman Center for Photomedicine, United States of America

## Abstract

The new concept of Immunogenic Cell Death (ICD), associated with Damage Associated Molecular Patterns (DAMPs) exposure and/or release, is recently becoming very appealing in cancer treatment. In this context, PhotoDynamic Therapy (PDT) can give rise to ICD and to immune response upon dead cells removal. The list of PhotoSensitizers (PSs) able to induce ICD is still short and includes Photofrin, Hypericin, Foscan and 5-ALA. The goal of the present work was to investigate if Rose Bengal Acetate (RBAc), a powerful PS able to trigger apoptosis and autophagy, enables photosensitized HeLa cells to expose and/or release pivotal DAMPs, i.e. ATP, HSP70, HSP90, HMGB1, and calreticulin (CRT), that characterize ICD. We found that apoptotic HeLa cells after RBAc-PDT exposed and released, early after the treatment, high amount of ATP, HSP70, HSP90 and CRT; the latter was distributed on the cell surface as uneven patches and co-exposed with ERp57. Conversely, autophagic HeLa cells after RBAc-PDT exposed and released HSP70, HSP90 but not CRT and ATP. Exposure and release of HSP70 and HSP90 were always higher on apoptotic than on autophagic cells. HMGB1 was released concomitantly to secondary necrosis (24 h after RBAc-PDT). Phagocytosis assay suggests that CRT is involved in removal of RBAc-PDT generated apoptotic HeLa cells. Altogether, our data suggest that RBAc has all the prerequisites (i.e. exposure and/or release of ATP, CRT, HSP70 and HSP90), that must be verified in future vaccination experiments, to be considered a good PS candidate to ignite ICD. We also showed tha CRT is involved in the clearance of RBAc photokilled HeLa cells. Interestingly, RBAc-PDT is the first cancer PDT protocol able to induce the translocation of HSP90 and plasma membrane co-exposure of CRT with ERp57.

## Introduction

The concept of tolerogenic apoptosis [1] has been integrated with that of immunogenic apoptosis or Immunogenic Cell Death (ICD) [2]. ICD plays a key role in cancer therapy since it induces tumor cells to undergo cell death concomitantly with the emission of a spatiotemporal-defined combination of Damage-Associated Molecular Patterns (DAMPs) decoded by the immune system to activate antitumor immunity, prerequisite for an effective long-term therapeutic success [3]. In fact, beside the list of the characteristics needed to consider a dead cells an ICD cell, the description of ICD is mainly related to an operational definition, and thus the definitive assurance of the ICD onset can be achieved by the *in* *vivo* vaccination experiments [4].

DAMPs, normally hidden within live cells, perform predominantly non-immunological functions and acquire immunomodulatory activities once secreted or surface exposed on dying or stressed/damaged cells [5]. DAMPs stimulate immune responses through “dialogue” with T lymphocytes, Natural Killer (NK) cells and Antigen Presenting Cells (APCs), i.e., macrophages, B lymphocytes and Dendritic Cells (DCs) [3]. DAMPs involved in the ICD are: surface exposed calreticulin (ecto-CRT) [6], [7], Heat Shock Protein 70 (ecto-HSP70) and 90 (ecto-HSP90) [8]–[10]; secreted ATP [11], [12]; passively released High Mobility Group Box 1 (HMGB1) and HPSs or chaperokines [11], [13], DNA [14], uric acid [15], S100 protein [16], sphingosin [17] and they can be categorized on the basis of the death process stage during their occurrence, the relocation place, the release mechanism, the origin and the mechanisms of action [18], [19].

Few conventional approved anticancer therapeutics, including radiotherapies (i.e., γ-irradiation) and chemotherapies (i.e., doxorubicin, mitoxantrone, oxaliplatin, cyclophosphamide, bortezomib) induce ICD. This ability is stressor-dependent and relies on the induction of Reactive Oxygen Species (ROS) production and Endoplasmic Reticulum (ER) stress [19]. Recently, it has been demonstrated that also PhotoDynamic Therapy (PDT) induces ICD in cancer cells [10], [11], [20]–[22]. PDT is a cytotoxic treatment based on the interaction between light, cell or tissue molecular oxygen and photosensitizing molecule (PhotoSensitizer or PS). The photodynamic reaction elicits ROS production [23] and consequent ROS-mediated cell death. The PS subcellular localization dictates the primary site of damage and the consequent outcome of the treatment, implying direct cell damage (apoptotic and/or autophagic and/or necrotic cell death) and secondary effects (damage to the vasculature and inflammatory reaction ending in the systemic immunity) [24]. Well characterized DAMPs involved in PDT response include HPS70 [10], [21], [25], [26], CRT [10], [11], [20], ATP [11] and HMGB1 [20]. In PDT, DAMPs exposure and/or release have been elicited by using Photofrin [20], [21], [25], [26], Hypericin [10], [11], meso-tetrahydroxylphenyl chlorine (MTHPS, Foscan) [27], and 5-aminolevulinic acid (5-ALA) [28] as PSs. Here, we evaluate if oxidative stress elicited by Rose Bengal Acetate-PDT (RBAc-PDT) induces in HeLa cells the *in* *vitro* biochemical distinctive properties of ICD such as relocalization, i.e., exposure and/or release, of DAMPs in order to make a prediction about the capacity of RBAc to trigger ICD. In fact, in our previous papers we have demonstrated that RBAc-PDT ensures in HeLa cells the rapid, independent and long-lasting onset of apoptosis and autophagy by several signalling pathways originating from or converging on almost all intracellular organelles (mitochondria, lysosomes, Golgi apparatus and ER), despite RBAc primary perinuclear localization [29]–[33]. In addition, we showed that 1) apoptotic and autophagic RBAc photokilled HeLa cells efficiently recruit macrophages; 2) macrophages efficiently phagocyte dead HeLa cells; and 3) macrophages following internalization release IL-10, TGF-β and TNF-α [34]. Thus, since phagocytosis represents a critical effector mechanism of the immune system, here we endorse the hypothesis of immunogenic elicitation *via* RBAc-PDT by evaluating surface-exposed and/or released DAMPs, i.e., HSP70, HSP90, CRT, ATP and HMGB1, in RBAc-PDT-induced apoptotic and autophagic HeLa cells.

## Materials and Methods

### Cells and photodynamic treatment

#### HeLa cells

Human cervical carcinoma HeLa cells were cultured in Eagle’s Minimum Essential Medium (EMEM) (Cambrex, Verviers, Belgium) supplemented with 10% Fetal Bovine Serum (FBS) (Cambrex, Verviers, Belgium), 2 mM L-glutamine (Cambrex, Verviers, Belgium), 100 IU/mL penicillin and streptomycin solution (Sigma, St. Louis, MO, USA) and 10000 U/mL amphotericin B (antimicotic solution) (Cambrex, Verviers, Belgium), in a 5% CO_2_ humidified atmosphere at 37°C. The cells were maintained in 75 cm^2^ flasks (between 5×10^5^ and 1×10^6^ cells/mL) by passage every 3 to 4 days.

#### Human macrophages

Human monocytes were isolated from the ‘buffy coats’ of healthy blood donors on Ficoll–Paque Plus (Amersham Biosciences, Glattbrugg, Switzerland) gradients and magnetic separation using CD14 human microbeads (Miltenyi Biotec, Auburn, CA, USA). Human macrophages were obtained through five day differentiation using 5 ng/mL macrophage colony-stimulating factor.

#### RBAc photodynamic treatment

For photodynamic treatment, HeLa cells were incubated with RBAc (10^−5^ M) in EMEM medium supplemented with 10% FCS for 60 minutes at 37°C as already reported [31]. Briefly, a stock solution (10^−2^ M) was obtained by diluting RBAc (Sigma-Aldrich, St. Louis, Mo, USA) in dimethyl sulfoxide. After incubation, the culture medium was replaced with phosphate buffer saline (0.2 M PBS, pH 7.4), previously allowed to equilibrate with 5% CO_2_ humidified atmosphere at 37°C, without phenol red, to avoid undesired photosensitizing effects. Cells were then exposed for 90 seconds to green light emitting diode, LED DPL 305, (QTL Inc., Atlanta, USA) emitting at 530±15 nm, in order to obtain 1.6 J/cm^2^ as total light dose. Cells were then rinsed twice with PBS 0.2 M pH 7.4, transferred to drug-free complete medium and allowed to recover for different times (from 1 to 24 h).

The photodynamic treatment was performed in the presence of apoptosis (pan-caspase inhibitor, Z-VAD-FMK, 20 µM; R&D Systems, Minneapolis, MN, USA), autophagy (3- MethylAdenine, 3-MA, 10 mM; Sigma-Aldrich Chem. Co., St Louis, MO, USA) and necroptosis (Necrostatin 1, Nec-1, 300 µM; Santa Cruz Biotechnology Inc., Santa Cruz, CA, USA) specific inhibitors in order to obtain autophagic and apoptotic cells fractions at 90% purity [30]. The autophagic HeLa cells fraction was obtained by using simultaneously Z-VAD-FMK and Nec-1; apoptotic HeLa cells fraction was obtained by using 3-MA and Nec-1. The cell death inhibitors were added 30 min before photodynamic treatment, during RBAc treatment (1 h incubation) and after irradiation during the recovery time periods (1–24 h), in a 5% CO_2_ humidified atmosphere at 37°C. Untreated cells (CNTR) are considered as control of experiments. Apoptotic and autophagic dead cells were evaluated by using the AnnexinV/PI (Sigma-Aldrich, St. Louis, MO, USA) and monodansylcadaverine (MDC) (FlukaChemie, Buchs, Switzerland) staining respectively.

#### Ethics Statements

Human blood samples were obtained by buffy coats supplied by the Hospital S. Giuseppe da, Lecce, Italy. Donors were anonymous to us. The need of donor consent was waived by the Ethics Committee. The use of buffy coat was acknowledged by the Comitato Etico dell’ASL LE, Lecce, Italy (Ethics Committee of the Health Service of Lecce). This Ethics Committee is an independent organization that is working under the Declaration of Helsinki and following the rules of Good Clinical Practices according to international and national laws and to the guidelines of the Italian National Committee of Bioethics. Ethics Statements Human blood samples were obtained by buffy coats supplied by the Hospital S. Giuseppe da Copertino, Lecce, Italy. Donors were anonymous to us. The need of donor consent was waived by the Ethics Committee. The use of buffy coat was acknowledged by the Comitato Etico dell’ASL LE, Lecce, Italy (Ethics Committee of the Health Service of Lecce). This Ethics Committee is an independent organization that is working under the Declaration of Helsinki and following the rules of Good Clinical Practices according to international and national laws and to the guidelines of the Italian National Committee of Bioethics.

### Immunoblotting analysis

At fixed times post PDT, purified plasma membrane proteins of HeLa cells were obtained according to the Plasma Membrane Protein Extraction Kit instructions (BioVision, Mountain View, CA, USA), while the culture medium (reported as Conditioned Medium, CM) in which the experiments were performed was collected and proteins were precipitated in acetone.

Determination of protein concentration and immunoblotting were performed as described in [30]. Immunoblotting was done with primary antibodies against HSP70 (1 µg/mL), HSP90 (1 µg/mL), CRT (1 µg/mL), ERp57 (20 µg/mL), HMGB1 (1 µg/mL; MBL, Woburn, MA, USA). Appropriate IgG Biotin-conjugated secondary antibodies (1 µg/mL) were purchased from Sigma-Aldrich Chem. Co. (St. Louis, MO, USA). The monoclonal e-cadherin (1 µg/mL; R&D Systems, Minneapolis, MN, USA) and β-actin primary antibody (1 µg/mL) (Sigma-Aldrich, St. Louis, MO, USA) were used as control.

The densitometer analysis was performed at GS-700 Imaging Densitometer (Bio-Rad, Hercules, CA, USA). The results were reported as ratio treated/untreated band intensity.

### Intracellular and cell surface CRT fluorescence detection

For immunofluorescence analysis, HeLa cells after RBAc photodynamic treatment were fixed in paraformaldehyde (4% in PBS 0.2 M, pH 7.4) for 5 minutes. For intracellular immunofluorescence detection the cells were permeabilized with Triton (0.1% in PBS 0.2 M, pH 7.4). Permeabilized and not permeabilized HeLa cells were incubated before with polyclonal anti-CRT developed in rabbit (1∶200 in PBS/BSA0.1%; Sigma-Aldrich, St. Louis, MO, USA) for 1 h at RT and then with the anti-rabbit IgG antibody FITC-conjugated (1∶25 in PBS/BSA 0.1%; Sigma-Aldrich, St. Louis, MO, USA) for 1 h at dark and RT. Fluorescence was evaluated with a Eclipse 80i fluorescence microscope (Nikon, Tokyo, Japan).

### ATP assay

At fixed times post PDT, extracellular ATP, measured in the conditioned media, and intracellular ATP were determined by using the Human Adenosine Triphosphate (ATP) Elisa kit (MyBioSource, San Diego, California, USA), and luciferin-based ENLITEN ATP assay (Promega, Madison, WI, USA) following manifacturer’s instructions. For ELISA assay, optical Density (O.D.) at 450 nm was determined by using the microplate reader Thermo electron corporation MULTISKAN EX ORIGINAL (Thermo Fisher Scientific Inc., Waltham, MA, USA). For luciferin-luciferase conversion assay, bioluminescence was assessed by using the microplate reader Thermo LabSystems Luminoskan Ascent luminometer (Thermo Fisher Scientific Inc., Waltham, MA, USA).

### Phagocytosis assay

Phagocytosis of autophagic and apoptotic HeLa cells was performed using human macrophages as phagocytes. Human macrophages were incubated with autophagic and apoptotic HeLa cells in a ratio of 10∶1 (10 dead cells per phagocyte) for 4 h at 37°C. Dead cells were stained with 1 mg/mL Hoechst 33342 for 5 min at 37°C before being added to the macrophage cultures. In inhibition experiments, chicken polyclonal anti-CRT (abcam, Cambridge, UK) added 30 minutes before phagocytosis assay was used. The phagocytosis rate (number of macrophages engulfing at least one autophagic or apoptotic cell) and phagocytosis index (number of autophagic or apoptotic cells internalized per macrophage) were reported by scoring at least 500 cells for each experiment, under a fluorescence microscope NIKON Eclipse 80i (Nikon, Tokyo, Japan), with Plan Fluor objectives (Nikon). The percentage of cells binding but not ingesting autophagic or apoptotic cells was calculated and expressed as percentage of binding.

### Statistical Analysis

The two-tailed Student’s t-test was used to analyze differences between controls and treated samples. Data are presented as mean value ±SD and all tests were performed at the 0.05 significance level.

## Results

### RBAc-PDT-induced death in HeLa cells

To determine the RBAc-PDT-induced death in HeLa cells, they were recovered for different periods after the PDT. Apoptosis and necrosis in the cell cultures were assessed by using the phosphatidylserine (PS)-binding Annexin V-FITC and the vital dye PI staining. The different cell deaths were discriminated on the basis of the labeling: apoptotic (Annexin V+/PI –) and necrotic (Annexin V+/PI+). Autophagic cells were discriminated by using monodansylcadaverine (MDC) and the vital dye PI staining and were identified as MDC+/PI – cells. The results are reported in Table I.

**Table 1:** Percentage of Annexin V, Propidium Iodite (PI) and Monodansylcadaverine (MDC) positive HeLa cells untreated and at different recovery times after incubation with Rose Bengal Acetate 10^−5^ M 1 h and irradiation with 1.6 J/cm^2^ 90 sec.

Recovery time (h)	Annexin V+/PI−			MDC+/PI−			Annexin V+/PI+		
	CTRL			CTRL			CTRL		
1	2,8±1,1	1,5±0,2	1,8±0,7	1,9±0,3	1,1±0,02	1,6±0,6	0,98±0,01	0,2±0,04	0,2±0,02
2	3,9±0,9	1,1±0,1	1,5±0,9	1,8±0,2	1,2±0,1	1,9±0,5	1,3±0,02	0,2±0,09	0,5±0,09
4	3,5±1,7	2,1±0,9	2,1±0,6	1,9±0,5	0,9±0,07	2,1±0,6	1,5±0,08	0,5±0,08	0,8±0,04
8	8,1±1,1	4,9±0,5	1,7±0,5	2,1±0,9	1,1±0,08	1,8±0,7	1,9±0,9	0,4±0,23	0,6±0,1
12	8,9±±0,98	6,7±1,1	1,9±0,6	2,4±0,7	0,7±0,04	2,1±0,3	2,1±0,7	0,38±0,15	0,38±0,15
18	10,4±0,4	7,3±±1,6	2,1±0,7	2,6±0,5	0,5±0,05	2,1±0,2	2,5±0,9	0,6±0,21	0,5±0,5
24	11,2±0,9	7,9±1,2	2,5±0,5	2,8±0,7	1,1±0,03	2,7±0,3	2,4±0,5	0,3±0,09	0,41±0,35
	**RBAc-PDT**			**RBAc-PDT**					
1	29,4±2,1*a	22,9±2,5*f	5,4±0,7*d	5,6±0,6*a	0,7±0,04e	5,2±0,8*a	1,2±0,09	0,3±0,03	0,29±0,02
2	28,9±1,9*a	23,1±1,7*f	8,1±0,9*d	8,5±0,7*b	0,7±0,02e	6,8±1,1*b	3,2±0,2	0,31±0,09	0,34±0,09
4	30,4±0,9*a	14,9±1,9*e	14,9±1,1*e	15,2±1,2*c	0,9±0,09e	14,9±1,3*c	5,1±0,05	0,41±0,1	0,39±0,17
8	41,5±1,4*b	15,9±1,1*e	24,9±1,2*a	25,2±1,1*d	1,1±0,02e	25,3±0,1*d	7,02±1,1	0,3±0,09	0,36±0,09
12	49,8±1,1*c	39,1±3,2*b	20,6±0,9*f	10,1±0,5*b	1,2±0,01e	9,8±0,1*b	8,1±0,54	0,39±0,19	0,33±0,19
18	42,6±0,9*b	33,8±3,4*a	21,3±1,1*f	9,5±1,6*b	0,9±0,03e	9,2±1,1*b	8,3±0,43	0,41±0,21	0,36±0,15
24	43,1±1,3*b	32,1±3,3*a	18,9±0,7*f	9,2±1,2*b	0,9±0,07e	9,1±0,2*b	9,1±1,2	0,37±0,21	0,33±0,2
z-VAD	−	−	+	−	−	+	−	−	+
3-MA	−	+	−	−	+	−	−	+	−
Nec-1	−	+	+	−	+	+	−	+	+

The fraction of apoptotic, autophagic and necrotic dead cells was determined by counting Annexin V+/PI−, MDC+/PI− and Annexin V−/PI+ cells respectively. At least 500 cells were scored for each time with a fluorescence microscope. Values are the average ± SD of three independent experiments. Asterisks show significant values (p<0.05) versus untreated ones. Significantly different (p<0.05) values among samples are indicated by different letters. HeLa cells were incubated with Rose Bengal Acetate for 60 min followed by 90 seconds light irradiation (1.6 J/cm^2^) and then by recovery in fresh medium (1, 2, 4, 8, 12, 18 and 24 h). Z-VAD-FMK: [Benzyloxycarbonyl-Val-Ala-Asp (OMe) fluoromethylketone] pan caspases inhibitor; 3-MA: [3-methyladenine] autophagy inhibitor; Nec-1: [Necrostatin-1] necrosis inhibitor.

Consistent with our previous studies, defined quantities of apoptotic, autophagic and necrotic cells are triggered at a determined time after PDT [31]. A recovery time-dependent increase of apoptosis and of autophagy of 40% at 12 h and of 25% at 8 h post irradiation respectively, was measured. PS exposure on Annexin V+/PI− cells is as early as 1 h after PDT; the number of Annexin V+/PI− cells increased with time and peaked at 12 h post irradiation. The increase of Annexin V+/PI+ cells was observed at the end of the recovery time, i.e. 24 h post RBAc-PDT as a consequence of secondary necrosis for the *in* *vitro* lack of clearance of dead cells. In fact, in our system necrosis was always negligible, less than 5%. The independent onset of apoptosis and autophagy allowed to obtain 90% pure autophagic and apoptotic cells fractions by using respectively the inhibitors z-VAD and Nec-1 or 3-MA and Nec-1.

### Ecto-CRT is accompanied by co-translocation of ecto-ERp57

**Figure 1:**
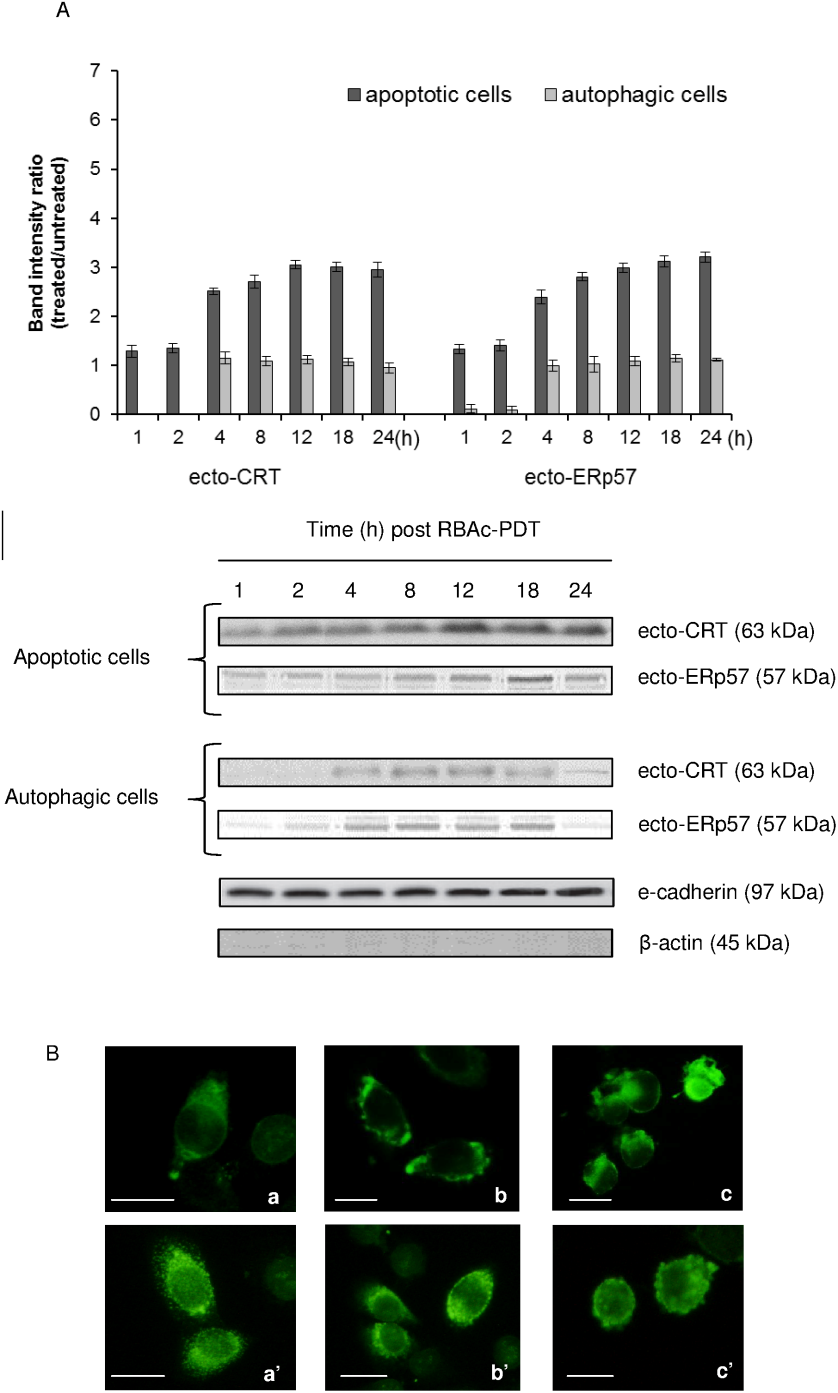
Surface exposure of CRT and ERp57 in RBAc-PDT-treated HeLa cells. **A.** Kinetic of CRT and ERp57 membrane translocation (ecto-CRT and ecto-ERp57) was performed by Western blot of electroblotted to nitrocellulose membrane SDS-PAGE of membrane proteins fraction (30 µg protein/lane) of RBAc-PDT treated HeLa cells (10^−5^ M RBAc, 1 h, 1.6 J/cm^2^, 90 sec) in the presence of 3-MA (10 mM) and Nec-1 (300 µ) (apoptotic cells) and in the presence of z-VAD-FMK (20 µM) and 3-MA (10 mM) (autophagic cells) at indicated time intervals. Monoclonal antibodies against CRT (63 kDa) and ERp57 (57 kDa) were used. The amount of CRT and ERp57 proteins was reported as band intensity ratio (treated/untreated) measured by densitometer analysis. E-cadherin (97 kDa) and β-actin (45 kDa) expression is shown as control. Actin is absent in the membrane protein fraction. The data are the mean ± SD of three independent experiments. The values of the apoptotic cells are always significantly different (p<0.05) with respect to autophagic cells. The values of apoptotic cells values are significantly different (p<0.05) with respect to untreated cells for both ecto-CRT and ERp57. Unless the short times after PDT (2 h) whose values are significantly different (p<0.05) with respect to untreated cells, for all the other time points, the values of ecto-CRT and ecto-ERp57 of autophagic cells are not different from untreated cells. One representative Western Blot is shown out of the three independent experiments performed. **B.** Fluorescence micrographs of non permeabilized (a–c) and permeabilized (a’–c’) HeLa cells immunostained for CRT: viable (a, a’); 8 h (autophagic cells) (b, b’) and 12 h (apoptotic cells) (c, c’) of recovery after RBAc-PDT. Bar = 10 µm.

Untreated HeLa cells, as other eukaryotic cells [35], display discrete amount of CRT localized in small clumps on cell surface (Fig. 1B, a). RBAc-PDT promotes the expression of CRT on plasma membrane of apoptotic but not autophagic photokilled HeLa cells (Fig. 1). In fact, the level of ecto-CRT on autophagic cells was never significant different from untreated cells. Only at the first two hours after PDT on autophagic cells there is the complete loss of ecto-CRT from the cell surface. In the pictures of permeabilized cells (Fig. 1B, a’–c’) the fluorescence is showing also the intracellular CRT. Ecto-CRT level increases very early after RBAc-PDT on apoptotic cell surface (1 h) and is threefold the control at 12–24 h (Fig. 1A). The localization pattern of CRT is as uneven patches on the apoptotic cells (Fig. 1B, c), and is like viable cells (Fig. 1B, a) on the autophagic ones (Fig. 1B, b).

Since data in literature suggest that immunogenic CRT cell surface translocation occurs in a complex with ERp57 in relation to cell death stressor, the level and kinetic of ERp57 translocation on plasma membrane of apoptotic and autophagic HeLa cells was evaluated (Fig. 1). Western Blots and densitometric analysis revealed a strong correlation between ecto-ERp57 and ecto-CRT exposure on the cell surface of apopotic cells. RBAc-PDT induces the translocation of ERp57 to the cell surface at levels and with a kinetic similar to CRT (Fig. 1). Ecto-ERp57 was not significantly exposed on the autophagic cells.

**Figure 2:**
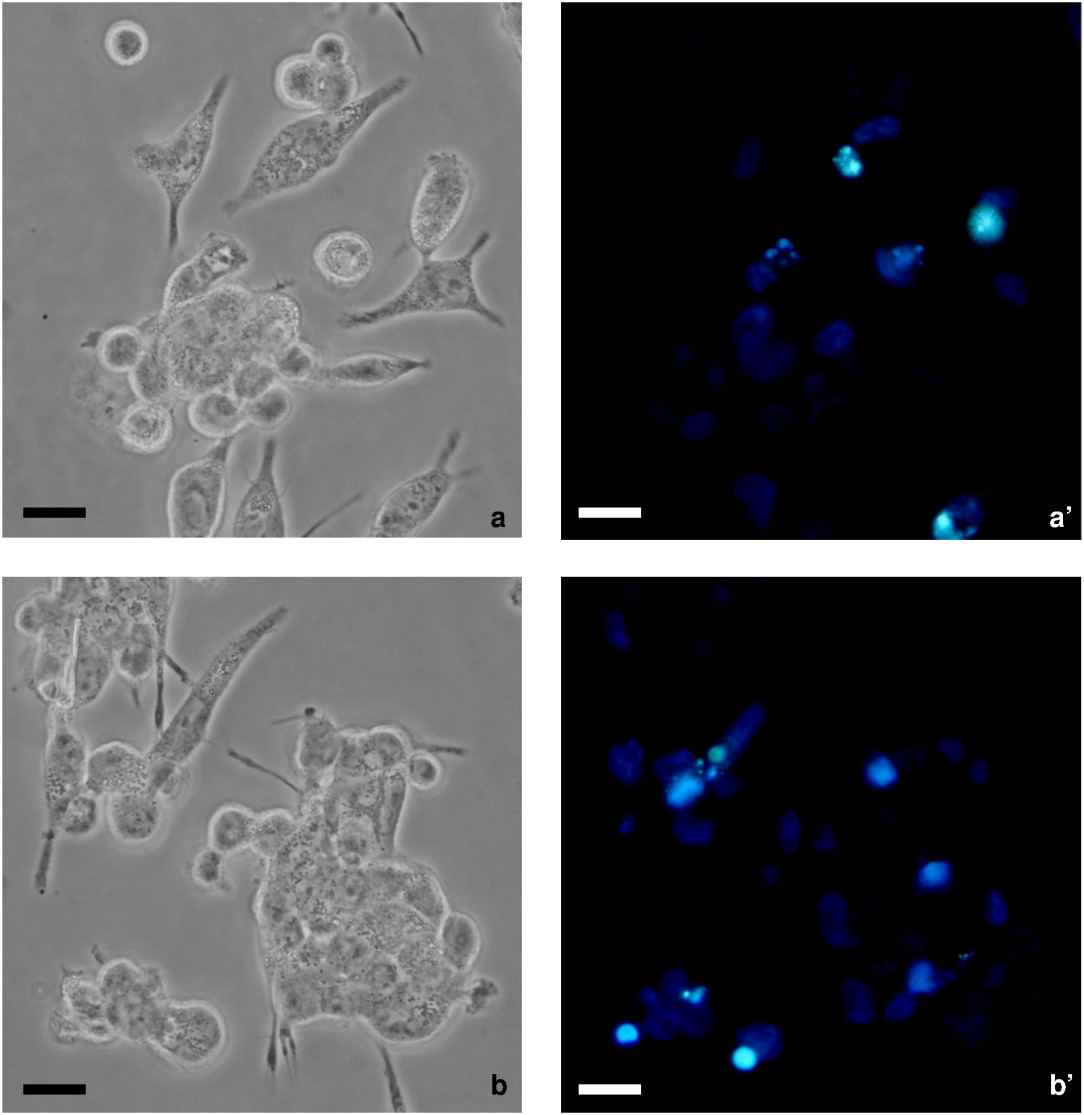
Involvement of ecto-CRT in phagocytosis of RBAc photosensitized HeLa cells. Phase contrast micrographs (a, b) and fluorescence micrographs of Hoechst (1 µg/mL) labeled (a’–b’) apoptotic RBAc-PDT-induced (RBAc 10^−5^ M 60 min followed by 90 seconds irradiation with green light (1.6 J/cm^2^) and then by recovery in fresh medium for 12 h, in the presence of 3-MA 10 mM and Nec-1 300) HeLa cells co-incubated with human macrophages. a–b) inhibition of ecto-CRT by polyclonal chicken anti-CRT. Bars = 10 µm.

Macrophages efficiently bind and ingest PDT treated HeLa cells with difference in the rate but not in the index of phagocytosis depending on the death type (Table 2). Macrophages preferentially tether, tickle and engulf apoptotic better than autophagic RBAc-PDT-induced HeLa cells. The phagocytosis rate and the percentage of binding are partially dependent on the exposure of ecto-CRT as the experiments of phagocytosis in the presence of anti-CRT antibody are showing (Table 2). In fact, ecto-CRT exposed on plasma membrane of apoptotic but not autophagic cells cooperates in clearance of dead cells, whose internalization is significantly reduced of about 50% by the specific anti-CRT antibody (Fig. 2 and Table 2).

**Table 2:** Phagocytosis index and rate, percentage of binding and number of apoptotic and autophagic HeLa cells bound per isolated human macrophages.

HeLa cells co-cultured with macrophages	Phagocytosis Index		Phagocytosis Rate (%)		Number of dead cells bound/macrophage		Binding (%)	
	–anti-CRT	+anti-CRT	–anti-CRT	+anti-CRT	–anti-CRT	+anti-CRT	–anti-CRT	+anti-CRT
Viable	N.D.	N.D.	N.D.	N.D.	N.D.	N.D.	N.D.	N.D.
RBAc-PDT (12 h rec)	1,878±0,024	1,865±0,101	25,106±0,017*	12,945±0,066	3,585±0,025	3,717±0,023	48,136±0,168*	24,136±0,141
RBAc-PDT (8 h rec)	1,754±0,007	1,754±0,018	15,774±0,083	16,322±0,004	3,464±0,061	3,507±0,180	39,505±0,008	40,099±0,111

Phagocytosis index: number of dead cells internalized per macrophage.

Phagocytosis rate: number of macrophages internalizing at least one dead cell.

Percentage of binding: number of macrophages binding at least one dead cell.

Viable: untreated HeLa cells; RBAc-PDT (12 h rec): apoptotic PDT-induced in the presence of 3-MA (10 mM) and Nec-1 (300 µ) HeLa cells, 12 h of recovery post PDT; RBAc-PDT (8 h rec): autophagic PDT-induced in the presence of z-VAD-FMK (20 µ) and 3-MA (10 mM) HeLa cells, 8 h of recovery post PDT.

N.D. Not Detected value, corresponding to 0 cells counted.

RBAc-PDT: HeLa cells incubated with Rose Bengal Acetate for 60 min followed by 90 seconds irradiation with green light (1.6 J/cm^2^) and then by recovery in fresh medium (8 and 12 h).

Each value is the average ±SD of 500 cells scored out of three independent experiments. Asterisks show significant (-anti-CRT) values (p<0.05) versus (+anti-CRT) ones.

### RBAc-PDT induces surface exposure and release of HSP70 and HSP90

**Figure 3:**
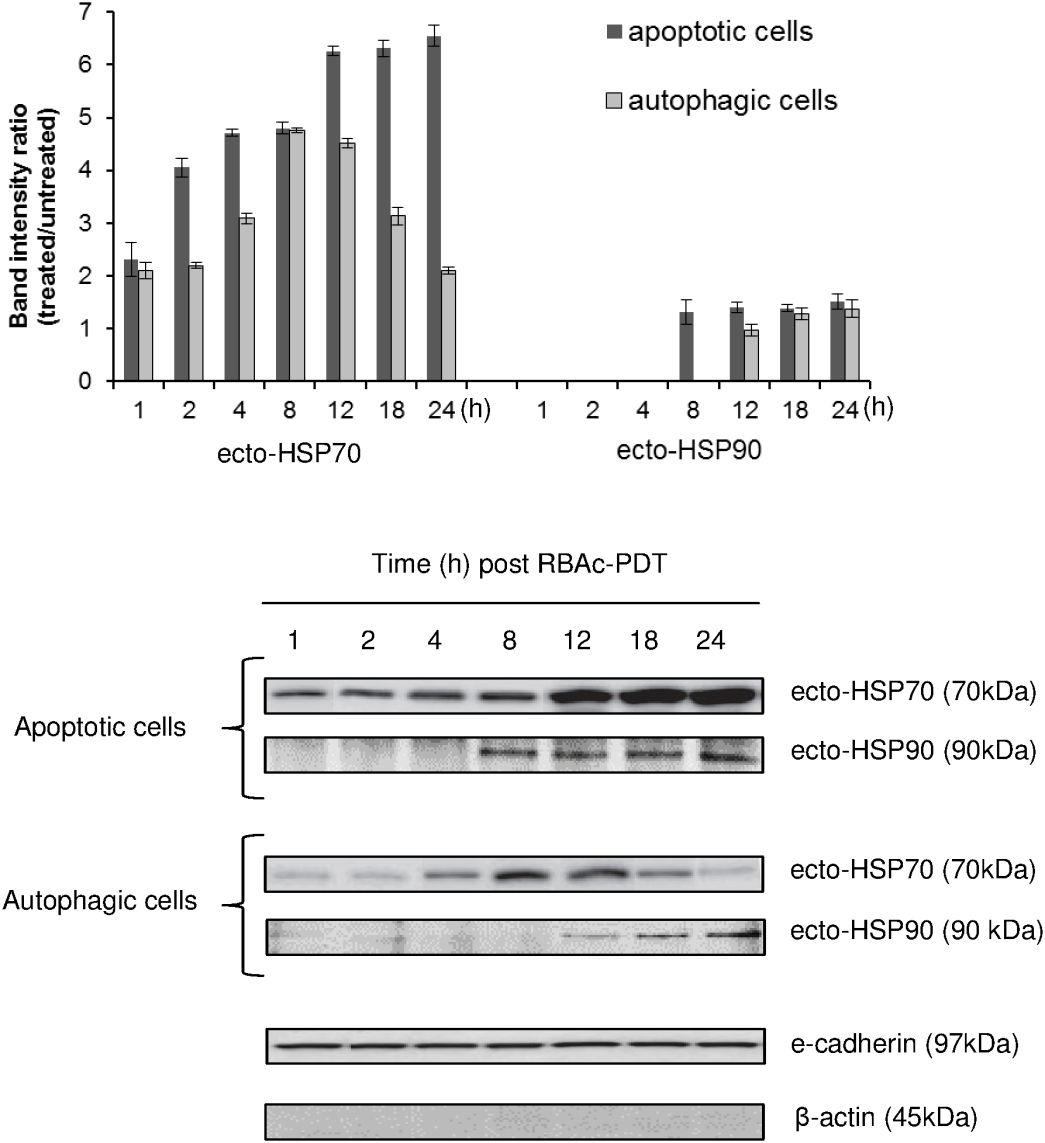
Surface exposure of HSP70 and HSP90 in RBAc-PDT-treated HeLa cells. Kinetic of HSP70 and HSP90 membrane translocation (ecto-HSP70 and ecto-HSP90) was performed by Western blot of electroblotted to nitrocellulose membrane SDS-PAGE of membrane proteins fraction (30 µg protein/lane) of RBAc-PDT treated HeLa cells (10^−5^ M RBAc, 1 h, 1.6 J/cm^2^, 90 sec) in the presence of 3-MA (10 mM) and Nec-1 (300 µ) (apoptotic cells) and in the presence of z-VAD-FMK (20 µM) and 3-MA (10 mM) (autophagic cells) at the indicated time intervals. Monoclonal antibodies against HSP70 (70 kDa) and HSP90 (90 kDa) were used. The amount of HSP70 and HSP90 proteins was reported as band intensity ratio (treated/untreated) measured by densitometer analysis. E-cadherin (97 kDa) and β-actin (45 kDa) expression is shown as a control. Actin is absent in the membrane proteins fraction. The data are the mean ± SD of three independent experiments. The values for ecto-HSP70 of the apoptotic cells are always significantly different (p<0.05) with respect to autophagic cells. The values of apoptotic and autophagic cells values are significantly different (p<0.05) with respect to untreated cells for both ecto-HSP70 and ecto-HSP90. One representative Western Blot is shown out of the three independent experiments performed.

**Figure 4:**
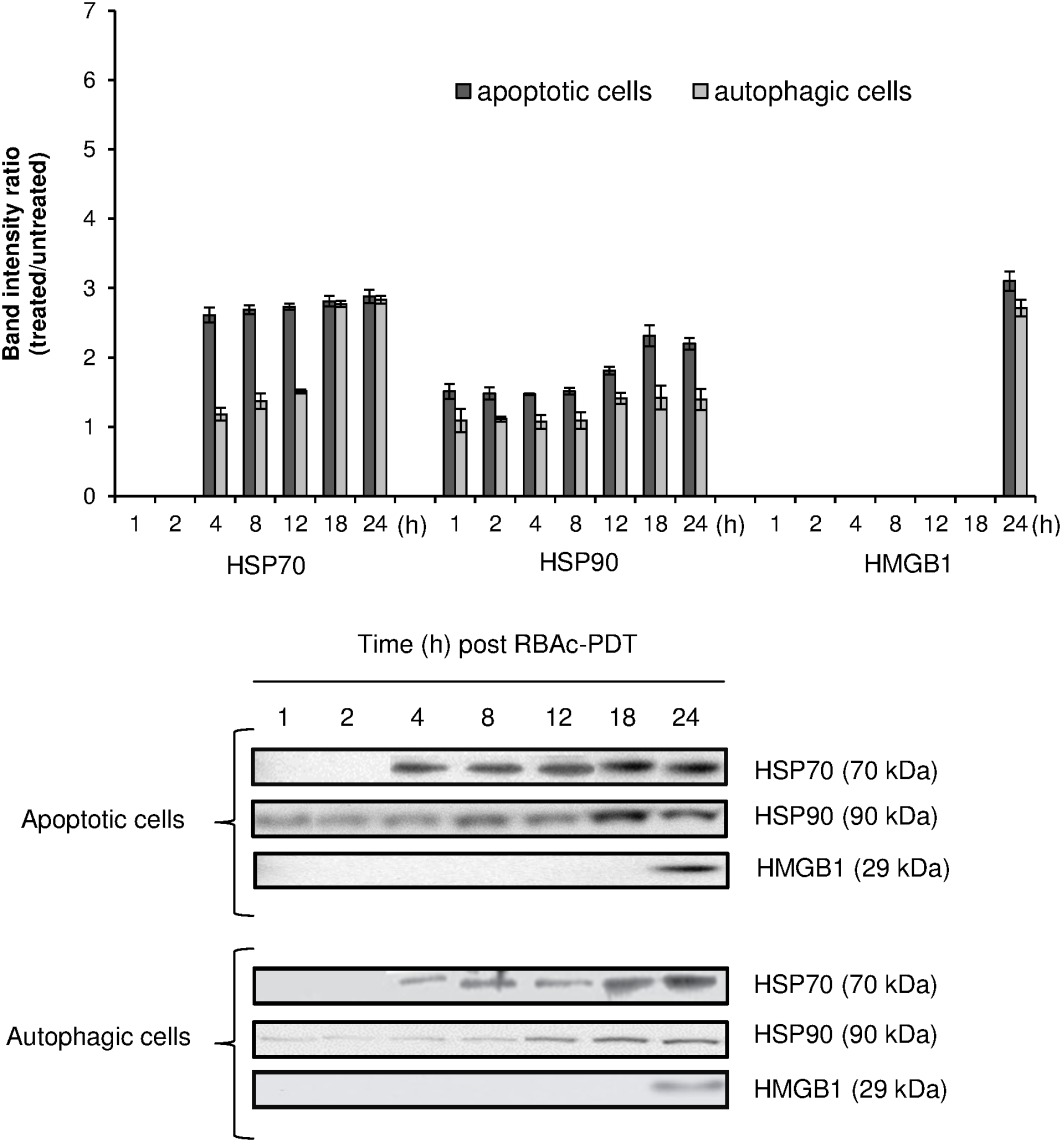
RBAc-PDT induces an early release of HSP70 and HSP90 and a late and passive release of HMGB1 in HeLa cells. Kinetic of released HSP70 and HSP90 was performed by Western blot of electroblotted to nitrocellulose membrane SDS-PAGE of the conditioned media proteins (30 µg protein/lane) of RBAc-PDT treated HeLa cells (10^−5^ M RBAc, 1 h, 1.6 J/cm^2^, 90 sec) in the presence of 3-MA (10 mM) and Nec-1 (300 µ) (apoptotic cells) and in the presence of z-VAD-FMK (20 µM) and 3-MA (10 mM) (autophagic cells) at indicated time intervals. Monoclonal antibodies against HSP70 (70 kDa), HSP90 (90 kDa) and HMGB1 (29 kDa) were used. The amount of HSP70, HSP90 and HMGB1 proteins was reported as band intensity ratio (treated/untreated) measured by densitometer analysis. Actin is absent in the conditioned media proteins. The data are the mean ± SD of three independent experiments. The values (4–12 h) of ecto-HSP70 of apoptotic cells are significantly different (p<0.05) with respect to autophagic cells. HSP90 and HMGB1values of apoptotic cells were always significantly different (p<0.05) with respect to autophagic cells. HSP70, HSP90 and HMGB1 values of apoptotic and autophagic cells were always significantly different (p<0.05) with respect to untreated cells. One representative Western Blot is shown out of the three independent experiments performed.

RBAc-PDT induces the translocation to the cell surface and the release in the conditioned media (CM) of HSP70 and 90 (Figs. 3 and 4). The early presence (1 h post RBAc-PDT) of a band of 70 kDa, corresponding to ecto-HSP70 was detected on isolated apoptotic and autophagic plasma membrane proteins. The amount of ecto-HSP70 on apoptotic cells ranges from about 2, 5 (1 h post PDT) to 6, 5 fold (12–24 h post PDT) the control untreated value. Level of ecto-HSP70 on autophagic cells peaked 8–12 h post PDT (4.5 fold the control untreated value).

The release of HSP70 and HSP90 was observed at 4 h and 1 h post PDT respectively (Fig. 4). Their release is not passive, since they were detected in the apoptotic and autophagic media at time after PDT in which the percentage of necrosis is negligible and secondary necrosis is not yet present. In general apoptotic cells release more HSP70 and 90 than autophagic ones with a maximum from 18 to 24 h post PDT; a significant release of HSP70 from autophagic cells was measured at 18–24 h (Fig. 4). HSP90 was released into culture medium early after RBAc-PDT (1 h after irradiation) and increased at longer recovery times (18–24 h for apoptotic cells and 12–24 h for autophagic ones) (Fig. 4).

### HMGB1 is passively and late released by HeLa cells RBAc-PDT treated

HMGB1 protein is not actively released into conditioned medium of autophagic and apoptotic RBAc-PDT-induced HeLa cells as reported in Fig. 4. HMGB1 levels rose significantly, threefold the value of untreated HeLa cells, only at 24 h after RBAc-PDT when secondary necrosis is largely present (Fig. 4).

### RBAc-PDT causes release of ATP in HeLa cells

**Figure 5:**
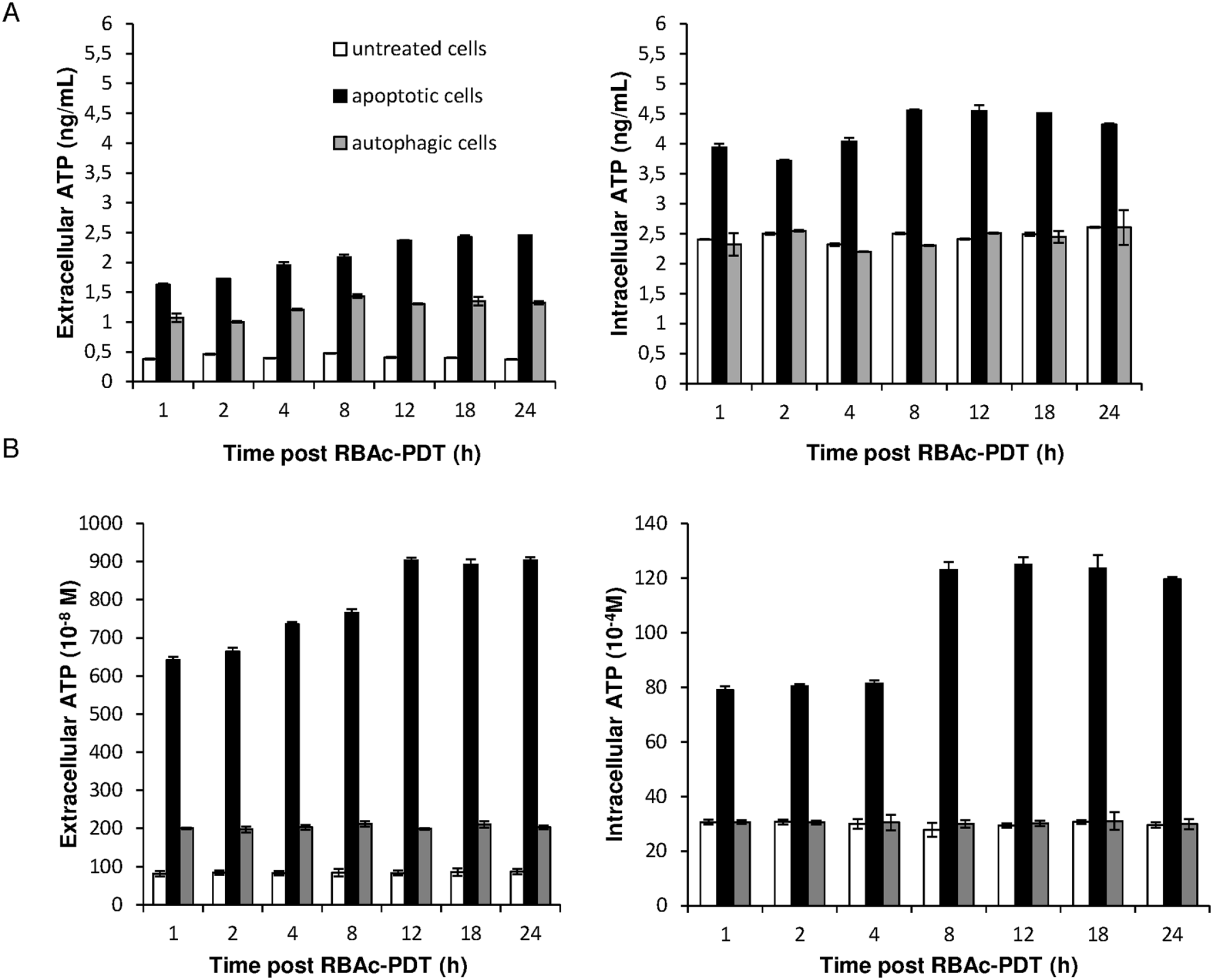
RBAc-PDT induces release of ATP in HeLa cells. Kinetic of intracellular and extracellular released ATP was performed by ELISA (A) and luciferin-luciferase based (B) assay on the conditioned media of RBAc-PDT treated HeLa cells (10^−5^ M RBAc, 1 h, 1.6 J/cm^2^, 90 sec) in the presence of 3-MA (10 mM) and Nec-1 (300 µ) (apoptotic cells) and in the presence of z-VAD-FMK (20 µM) and 3-MA (10 mM) (autophagic cells) at indicated time intervals. The amount of ATP, reported as ng/mL or as concentration (M), is the mean ± SD of three independent experiments. The values of extracellular ATP both of apoptotic and autophagic cells are significantly different (p<0.05) with respect to untreated cells. Extracelluar ATP values of apoptotic cells were always significantly different (p<0.05) with respect to autophagic cells. Intracellular ATP values of apoptoticc cells were always significantly different (p<0.05) with respect to autophagic and untreated cells.

RBAc-PDT treated HeLa cells secreted ATP into their culture medium under non-permeabilizing plasma membrane conditions soon after 1 h post irradiation (Fig. 5). The measurements were done by using two different methods (ELISA assay and luciferine-luciferase conversion assay) tha gave the comparable results (see figure 5 A *vs* B). The amount of ATP released into the medium was higher for the apoptotic than the autophagic cells. Indeed, while autophagic cells secreted the same amount of ATP during the 24 h of recovery, a progressive increase of release of ATP from apoptotic cells was observed.. In addition, in RBAc-PDT-induced, The intracellular ATP content of apoptotic cells rose considerably during the entire recovery, showing a further increase from the 8 h of post irradiation (Fig. 5). Conversely the intracellular content of ATP of autophagic cells was never different from the control ones.

## Discussion

We recently reported the efficient induction of ROS-mediated apoptosis and autophagy in HeLa cells upon RBAc-PDT [31]. HeLa photokilled cells actively drive their *in* *vitro* and *in* *vivo* fast clearance by macrophages through the production of “eat me” and “find me” signals [34]. In fact, photokilled HeLa cells recruit, tether and tickle macrophages that consequently release TNF-α, TGF-β and IL-10, thus premising the immunomodulatory effects of RBAc-PDT [34]. To deeply clarify the RBAc-PDT immunomodulatory effects, we investigated on its ability to induce exposure and/or release of DAMPs, the molecules eliciting the ICD [4], [5]. Like other PSs utilized in PDT protocols, i.e. photofrin [20], [21], [25], [26], hypericin [10], [11], Foscan [27], and 5-ALA [28] RBAc induces in HeLa cells some features that are characteristic of ICD. The release and/or exposure of DAMPs repertoire by RBAc-PDT is a valid requisite to predict that ICD could be induced. However, this hypothesis needs validation by *in* *vivo* performing vaccination experiments. The recent attraction that is receiving PDT relays on its ability to induce large extent of cell death and f to trigger ICD [5]. This is in line with the most recent therapeutic protocols that are aimed to combine the efficient induction of cell death with an adequate anti-tumor immunogenic response, most likely *via* ICD to overcome the two fateful hallmarks acquired by tumor cells: resistance to cell death and immunosurveillance overruling.

This work shows that RBAc photodynamic treatment promotes the plasma membrane translocation and the extracellular release of some DAMPs in both apoptotic and autophagic HeLa cells, but with differences related to type (CRT, HSP70 and HSP90 for apoptotic cells, HSP70 and HSP90 for the autophagic cells) and quantity (apoptotic > autophagic). Noteworthy, these data represent the first demonstration of the exposure of HSP90 upon PDT and of the exposure/release of DAMPs also in autophagic cell death induced *via* PDT. In fact, exposure of HSP90 was shown only in lung cancer [36] and myeloma [37] treated with Bortezomib and in bladder cancer cells treated with capsaicin [38].

Already at 1 h after PDT high amounts of CRT, distributed on the cell surface as unevenly patches, are exposed on apoptotic cells, suggesting that CRT exposure in our PDT system is an early event concomitant with phosphatidylserine (PtdS) exposure on plasma membrane that mark the cells as apoptotic. It is known from the literature that the property of ICD dictates a preapoptotic exposure of CRT to make a death mechanism as immunogenic [39]. This is in contrast with our data showing CRT and PS exposure. This could be due to an early after PDT onset of apoptosis and to our experimental design in which 1 h after irradiation represents the earliest time of investigation. However, our result is in line with that observed for others ICD inducers, i.e., shikonin and coxsackievirus B3 (for a deep review the reader can see Krysko et al., 2012 [3]). Here, the role of CRT in removal of dead cells was also investigated. In fact, it is well known that the peculiar patchy distribution of ecto-CRT plays an important role in the phagocytosis-modulating efficacies and outcomes. Ecto-CRT, by co-localizing with PtdS on plasma membrane [40], functions as an “eat me” signal to favor phagocytosis in different cell lines such as fibroblasts, neutrophils and Jurkat T cells of dead cells by dendritic cells and macrophages that, consequently, initiate immune response [6], [41]. The CRT-PtdS domains on the apoptotic cells surface are very efficient “eat-me” signals that stimulate phagocytosis by segregating away the “don’t eat me” CD47 signals and by activating the internalization receptors, LRP (LDL-Receptor-related Protein, also known as CD91 receptor), present on phagocytes. [40]. This explains why macrophages do not internalize viable cells even when CRT is exposed. Indeed, the absence of PtdS on plasma membrane of viable cells impairs segregation of CD47 and thus phagocytosis of viable cells is blocked. In our system also ecto-CRT cooperates in the removal of apoptotic RBAc-PDT-induced HeLa cells as demonstrated by about 50% phagocytosis rate decrease by inhibiting ecto-CRT on plasma membrane. We already demonstrated that PtdS localized on plasma membrane of RBAc-PDT apoptotic HeLa cells with a temporal kinetic similar to that of ecto-CRT [30] and that both apoptotic and autophagic cells were efficiently recognized and phagocytosed by macrophages *via* modification of plasma membrane exposed glycans [34]. Ecto-CRT is, here, included in the list of ligand present on PDT-induced apoptotic cells plasma membrane dictating recognition and tethering by macrophages.

Data in literature suggest that the translocation of CRT on plasma membrane can occur concomitant or not to translocation of ERp57, ER-resident disulfide isomerase, in relation to stress inducer. In agreement with what already demonstrated by using anthracyclines, e.g. MTX, [42], in our system ERp57 was co-exposed with ecto-CRT at early apoptotic stage. Conversely, Garg et al., [10] demonstrate the ERp57-independent CRT exposure in PDT based on Hyp photosensitization. Thus, since several CRT translocation pathways have been described [10], [42], it would be interesting to investigate whether the different PSs induce the externalization of CRT following different molecular pathways. HSP70 externalization induced by RBAc-PDT is in line with data in literature reporting results obtained with different PSs, such as Hyp- [10], Photofrin- [21], [25], [26], Foscan [27], and 5-ALA [28]. In RBAc-PDT externalization of HSP70 is an early event (1 h) after irradiation and it is independent on the cell death type (apoptosis or autophagy); however, the amount of translocated HSP70 is greater in apoptotic cells than autophagic ones suggesting cell death type dependency.

To our knowledge, RBAc-PDT is the first PDT cancer protocol that induces translocation of HSP90. This translocation could be an advantage for anti-tumor therapy and make RBAc-PDT very attractive. In fact, it is known that exposure of HSP90 on the cell surface of dying cells is a mechanism for tumor cells adhesion to Dendritic Cells (DCs) as well as HSP70 on plasma membrane constitutes a mechanism for tumor antigen chaperoning that fight the tolerance of the immune system towards cancer cells [43]. Thus, RBAc-PDT *via* HSPs could likely elicit an immune response by ensuring the recognition of HeLa cancer cells by DCs as well as by macrophages [34].

ATP is released in the culture medium of RBAc-PDT-induced dead HeLa cells. As others well established ICD inducers i.e., MTX, oxaliplatin, UVC irradiation, γ-irradiation, anthracyclines and cardiac glycosides [3], RBAc-PDT induces an early release of ATP in culture medium of apoptotic cells. Interestingly, we demonstrates for the first time the release of ATP also in photodynamically-induced autophagic dead cells. The second line of hallmarks required for ICD is the late extracellular release of HMGB1, HSP70, and HSP90. In fact, the immunogenic potential of HSPs is more efficient when these molecules are released by apoptotic cells in the extracellular environment [44]. HMGB1 released as DAMPs is widely demonstrated in necrotic cancer cells [45] and only recently has been reported that apoptotic [44] and autophagic [46] cancer cells might release HMGB1 at some points in their execution phases. Data in literature report that Photofrin- [21], [25], Foscan- [27] and 5-ALA- [28] PDT induces the late HSP70 release, suggesting that the presence of the HSP70 in extracellular environment release is a consequence of membrane permeabilization following necrosis occurrence. In our system we observe an early to late release of HSP70 in apoptotic cells culture; conversely HSP70 is released mid to late in autophagic cells. In addition, HSP90 was precociously released (1 h after RBAc-PDT) by apoptotic HeLa cells whereas a mid to late release of HSP90 has been observed in RBAc-PDT-induced autophagic cells, suggesting the potentiality of the treatment to alert the immune system, ensuring cancer cells eradication. In fact, extracellular HSPs promote the formation of tumor antigen-HSP complexes that are processed by APCs for T cell cross-priming more efficiently that tumor antigen alone [5], [43], [47].

In agreement with literature data, the detection of HMGB1 into the culture medium coincided with the presence of secondary necrotic cells (24 h after RBAc-PDT) [31], suggesting a passive release of this DAMPs. Recently, Korbelik and coworkers demonstrated in mice serum with Lewis Lung Carcinoma (LLC) tumors treated with Photofrin, that actively secreted and passively released HMGB1 are respectively involved during the early and late stages after treatment [20].

In conclusion, our findings provide the evidence that RBAc-PDT induces relocalization of molecules known to be immunogenic, such as CRT, HSP70 and 90 and ATP. We also demonstrate that CRT in our system contributes to phagocytosis of RBAc-PDT-induced apoptotic cells. Altogether these data can be considered as surrogate ICD biomarkers whose immunogenic potential have to be further investigated by perform *in* *vivo* experiments. Indeed, the gold-standard approach to detect if RBAc-PDT is a ICD relies on vaccination experiments on immunocompetent murine models.

The fact that RBAc-PDT produces apoptotic and autophagic cells, both exposing and releasing DAMPs, makes RBAc-PDT a very promising immunogenic cancer modality that could contribute to increase the small arsenal of ICD inducers, thereby contributing to improve therapeutic PDT strategy by harnessing the immunogenicity of cancer cells.
